# Cervical artery dissection—an easily neglected cause of stroke: a case report

**DOI:** 10.1186/s12883-020-02006-6

**Published:** 2020-11-25

**Authors:** Ya -Hui Lian, Xin Chen, De- Rui Kong, Wei Chen, Ming-Chao Shi, Hong-Wei Zhou

**Affiliations:** 1 Department of Radiology, The First Hospital of Jilin University, Xinmin St. #71, Changchun, 130021 Jilin China; 2 Department of Neurology, The First Hospital of Jilin University, Changchun, Jilin China

**Keywords:** Cervical artery dissection, Stroke, 3D-T1 VISTA, VW-MRI

## Abstract

**Background:**

In recent years, the incidence of stroke has gradually increased in young people. There are many reasons causing stroke, including atherosclerosis, artery embolization, and cervical artery dissection and so on. However, cervical artery dissection is a major cause of stroke in young people. We present a case of ischemic stroke caused by dissection, whose distal vascular occlusion due to detachment of the thrombosis in the right internal carotid artery.

**Case presentation:**

A 33-year-old male patient was admitted to the hospital because of stroke. Imaging examination showed that there was no visualization of the right middle cerebral artery and there were a large number of mural thrombus in the C1 segment of the right internal carotid artery. After emergency surgery, the patient had vascular recanalization and the symptoms were significantly improved. Magnetic resonance imaging showed a high signal in the C1 segment of the right internal carotid artery, the abnormal signal disappeared after antiplatelet therapy.

**Conclusions:**

When a patient has symptoms of stroke, we need to explore the root cause of stroke. Especially in young people, cervical artery dissection is an important reason that can’t be ignored. Through review and analysis of this case, we hope to improve the understanding of radiologists and clinicians about the cervical artery dissection, reduce the rate of misdiagnosis, and improve patients’ prognosis.

## Background

Cervical artery dissection(CAD) includes internal carotid artery dissection(ICAD) and vertebral artery dissection(VAD). According to the literature report, VAD is a frequent cause of stroke in young patients [1]. Approximately 1% to 2% of all ischemic strokes are caused by CAD, but they produce up to 25% of ischemic strokes in young individuals [2]. CAD is characterized as a hematoma within the wall of the artery, the hematoma and the thrombus can lead to cerebral thromboembolism, decreased blood flow, and subsequent ischemic stroke [3], most of these strokes occur in the original position of the dissection. Here, we report a case of internal carotid artery dissection due to intimal damage, local thrombosis and thrombus shedding, resulting in distal middle cerebral artery occlusion.

## Case presentation

**Fig. 1:**
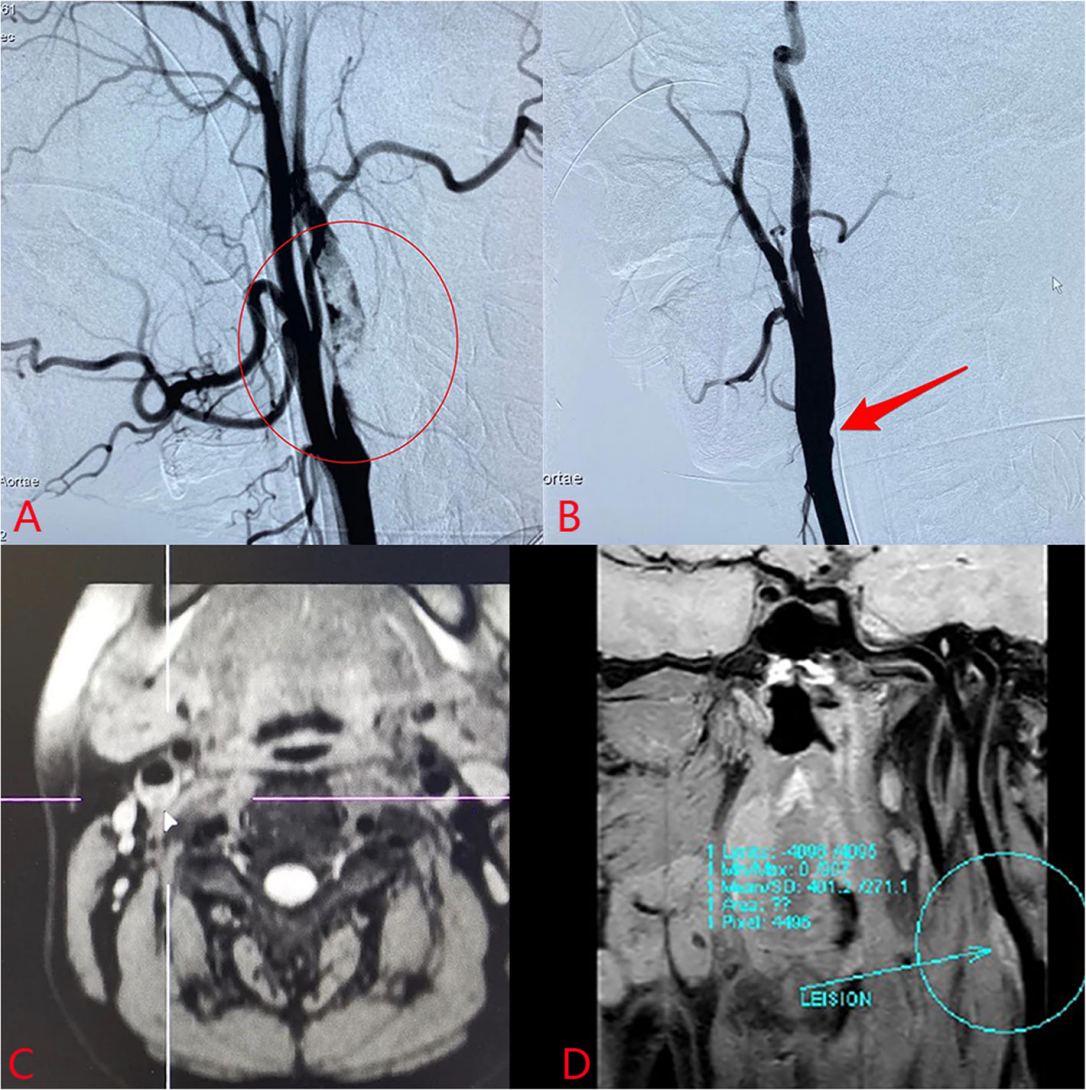
**a**: Digital subtraction angiography (DSA) showed a large number of mural thrombus in the C1 segment of the right internal carotid artery (red circle). **b**: Follow-up digital subtraction angiography showed the vessel wall of the right internal carotid artery was not smooth (red arrow). **c**: Axial 3D-T1 VISTA image showed a high signal in the lumen of the right internal carotid artery (white arrow). **d**: Curved planner reconstruction image showed the intramural hematoma of the right internal carotid artery (circle)

**Fig. 2:**
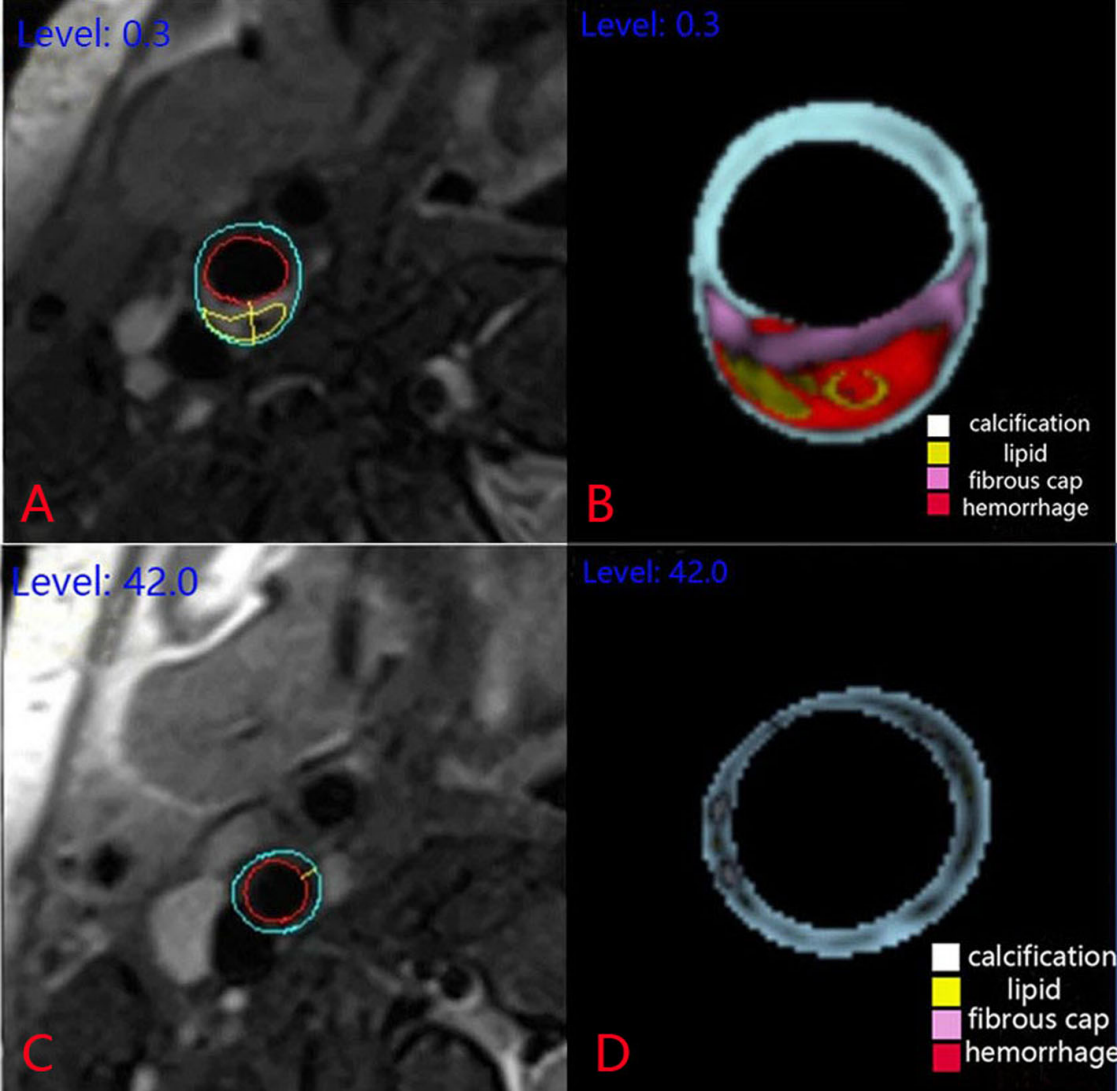
**a** and **b**: Before antiplatelet therapy, the right internal carotid artery was selected as the target lumen, and at the same level, the vessel wall magnetic resonance imaging showed a large number of thrombus and lipid core can be seen in the wall of the tube, it means that the mural thrombus is unstable plaque. **c** and **d**: After antiplatelet therapy, the right internal carotid artery was selected as the target lumen, and at the same level, the vessel wall magnetic resonance imaging showed the disappearance of abnormal components in the lumen

A 33-year-old male patient was admitted to the hospital because of ‘the numbness of the left upper limb for 3 h with dyskinesia of the left limb for 50 min’. The patient had a history of hypertension, smoking, and drinking. The physical examination showed that the muscle strength of the left upper limb reached 0 grade and the muscle strength of the left lower limb reached 3 grades. Digital subtraction angiography (DSA) showed a large number of mural thrombus in the C1 segment of the right internal carotid artery(RICA) (Fig. 1a). After the transcatheter thrombus aspiration and stent thrombectomy, the patient’s blood vessels were recanalized, while the vessel wall of the RICA was not smooth (Fig. 1b). By now, the patient's symptoms have been significantly improved. Four days after operation, the three-dimensional (3D) T1 volumetric isotropic turbo spin echo acquisition (VISTA) sequence showed a high signal in the lumen of the RICA (Fig. 1c). Curved planner reconstruction(CPR) showed a high signal in the vessel wall of the RICA (Fig. 1d). The vessel wall magnetic resonance imaging (VW-MRI) showed the components of hematoma in the vessel wall (Fig. 2a). One month after antiplatelet therapy (oral aspirin 100 mg once a day and oral clopidogrel 75 mg twice a day), VW-MRI showed the disappearance of hematoma in the vessel wall (Fig. 2b). So far, the patient is in good condition and is subject to regular follow-up. clopidogrel.

## Discussion

Cervical artery dissection(CAD) is the most common cause of ischemic stroke in young people. Dissection occurs when the intima or the media of the arterial wall is disrupted, causing an intramural hematoma in the subintimal, in the medial, or in the subadventitial layers [4]. Stroke in CAD is a consequence either of embolism originating from the injured intima or of hemodynamic compromise [5]. The typical presentation of CAD includes neck pain, headache, Horner syndrome(oculosympathetic palsy) and subsequent ischemia [6]. In our case, the patient does not show the typical clinical symptom of pain, and the first symptom is ischemic stroke, so it is difficult for us to make an accurate diagnosis of CAD.

The patient has long-term smoking, mild hypertension, hyperhomocysteinemia and dyslipidemia, which are common vascular risk factors in young stroke patients. In the postoperative DSA, the blocked blood vessels were recanalized, and the vessel wall of the C1 segment of the right internal carotid artery was not smooth, but the degree of lumen stenosis was mild. The 3D-T1 VISTA sequence shows the intramural hematoma of the right internal carotid artery, and we infer that dissection is the root cause of a large number of mural thrombus in this patient. When dissection occurs, thrombogenic factors are released by intimal damage, leading to local thrombus formation [7], the unstable plaque gradually enlarges and falls off, causing distal artery occlusion. Traditionally, DSA is considered the golden standard for dissection diagnosis due to the precise delineation of the lumen abnormalities [8]. Intimal flap, double lumen and “flame” sign are common finding in DSA as a result of arterial dissection. Unfortunately, these signs can be observed in less than 10% of CAD cases [9]. Furthermore, this technique does not provide any information about the artery wall. When dissection does not cause any alteration in the arterial lumen, it may lead to false negative in patients with CAD. A study shows that the 3D-T1 VISTA sequence is useful in the diagnosis of CAD it can show intramural hematoma of the cervical artery clearly [10]. It is worth pointing out that VW-MRI can not only display the lumen, but also image the vessel wall, it is helpful for further analyze the composition of plaque, judge the cause of stenosis and provide a direct imaging basis for clinical diagnosis and treatment. This suggests that VW-MRI plays an important role in the differential of various vasculopathies. In recent years, antithrombotic therapy and endovascular therapy have been widely used in clinic, the treatment choice is mainly based on the best judgment of the physician, considering the risks and benefits to the individual patient. Some studies have shown that endovascular therapy (EVT) has become the gold standard for treatment of proximal intracranial occlusions [11], it can achieve the recanalization of intracranial vessels as soon as possible. Antithrombotic therapy with either antiplatelets or anticoagulants is the mainstay of treatment for patients with CAD-related ischaemic stroke [12]. In our case, the patient was treated with endovascular treatment on admission, and the symptoms were significantly improved, subsequently, after one month of antiplatelet therapy, the abnormal signal in the wall of the RICA disappeared. In general, CAD is rarely determined as the stroke etiology within a few hours of disease progression, the diagnosis of the disease may be challenging due to the diversity of clinical manifestations, early identification of CAD and proper treatment can improve the prognosis of patients and reduce mortality.

## Data Availability

All data related to this case report are documented within this manuscript.

## Abbreviations

CAD: Cervical artery dissection
ICAD: Internal carotid artery dissection
VAD: Vertebral artery dissection
DSA: Digital subtraction angiography
RICA: Right internal carotid artery
CPR: Curved planner reconstruction
3D-T1 VISTA: Three-dimensional T1 volumetric isotropic turbo spin echo acquisition
VW-MRI: The vessel wall magnetic resonance imaging
EVT: Endovascular therapy
